# The Health Beneficial Properties of *Rhodomyrtus tomentosa* as Potential Functional Food

**DOI:** 10.3390/biom9020076

**Published:** 2019-02-21

**Authors:** Thanh Sang Vo, Dai Hung Ngo

**Affiliations:** 1 NTT Institute of Hi-Technology, Nguyen Tat Thanh University, Ho Chi Minh City 700000, Vietnam; 2 Faculty of Natural Sciences, Thu Dau Mot University, Thu Dau Mot City 820000, Binh Duong Province, Vietnam

**Keywords:** *R. tomentosa*, bioactivity, phenolic compound, terpenoid, functional food

## Abstract

*Rhodomyrtus tomentosa* (Aiton) Hassk. is a flowering plant belonging to the family Myrtaceae, native to southern and southeastern Asia. It has been used in traditional Vietnamese, Chinese, and Malaysian medicine for a long time for the treatment of diarrhea, dysentery, gynecopathy, stomachache, and wound healing. Moreover, *R. tomentosa* is used to make various food products such as wine, tea, and jam. Notably, *R. tomentosa* has been known to contain structurally diverse and biologically active metabolites, thus serving as a potential resource for exploring novel functional agents. Up to now, numerous phenolic and terpenoid compounds from the leaves, root, or fruits of *R. tomentosa* have been identified, and their biological activities such as antioxidant, antibacterial, anti-inflammatory, and anticancer have been evidenced. In this contribution, an overview of *R. tomentosa* and its health beneficial properties was focused on and emphasized.

## 1. Description of *Rhodomyrtus tomentosa* (Aiton) Hassk.

*R. tomentosa* is flowering plant in the family of Myrtaceae. It is mainly found in Southeast Asian countries, especially southern parts of Vietnam, China, Japan, Thailand, Philippines, and Malaysia. The leaves are opposite, 5–7 cm long and 2–3.5 cm wide, three-veined from the base, oval, obtuse to sharp pointed at the tip, glossy green above, densely grey, or rarely yellowish-hairy beneath, with a wide petiole, and an entire margin. The flowers are solitary or in clusters of two or three, 2.5–3 cm in diameter, with five petals which are tinged white outside with purplish-pink or all pink. The fruit is an ellipsoid berry that measures 1–1.5 cm in diameter with a persistent calyx. Unripe fruits have a green skin and an astringent taste. They turn to a purplish black color when ripe with the pulp being purplish in color, soft, and sweet. There are many deltoid seeds that measure 1.5 mm in diameter and are located in six (to eight) pseudo-locules, divided by thin false septa [1,2]. 

## 2. Ecology

*R. tomentosa* grows in moist and wet forests up to 2400 m elevation, on poor sand soils. It tolerates full sun and flooding. Moist, somewhat acid soils are preferred. The plant is not well adapted to limestone soils. It is able to invade a range of habitats, from pine flatwoods to mangrove marshes. It grows in a wide range of soil types, including salty coastal soil, but is sensitive to heavy salt spray. It is fire adapted, that is, able to resprout prolifically after fire. It has the potential to alter the natural fire regimes of invaded areas [3]. 

## 3. Nutritional Composition of *R. tomentosa* Fruits

The nutritional properties of *R. tomentosa* including proteins, amino acids, carbohydrates, lipids, vitamins, and minerals have been determined and reported [4,5,6]. It was found that *R. tomentosa* fruits contain the total protein of 4.00 ± 0.12% distilled water (DW). Moreover, they contain various amino acids, especially tryptophan, a precursor for the synthesis of serotonin, which is involved in mood, behavior, and cognition. Moreover, *R. tomentosa* fruit was found to have a remarkably high concentration of total dietary fiber (66.56 ± 2.31% DW). Soluble dietary fiber (SDF) represented only 7.60% of the total dietary fiber content. Most insoluble fibers found in *R. tomentosa* fruit were cellulose, which contributed to about 50% of the insoluble dietary fiber. Unlike dietary fiber, the digestible sugar content of *R. tomentosa* fruit was not high (19.96% DW) as compared with that of other tropical fruits. Besides, *R. tomentosa* fruit contains a low level of lipids (4.19 ± 0.07% DW). The most abundant fatty acids in *R. tomentosa* fruit were linoleic and palmitic acids, which contributed to 75.36% and 10.45% of total fatty acids, respectively. 

On the other hand, the analysis of *R. tomentosa* fruit has shown a clear observation regarding minerals and vitamins. It contains different minerals with high level of potassium (221.76 mg/150 g fruit), calcium (73.65 mg/150 g fruit), manganese (3.23 mg/150 g fruit), iron (1.54 mg/150 g fruit), zinc (0.61 mg/150 g fruit), and copper (0.40 mg/150 g fruit). Meanwhile, the vitamin C content of *R. tomentosa* fruit (5.62 mg/150 g fruit) was much lower than that of other tropical fruits, and the vitamin E level (3.89 mg/150 g fruit) was higher than that of mango and avocado [4]. 

## 4. Phytochemical Composition

*R. tomentosa* has been reported to contain various phytochemical compositions in many parts of the plant (Table 1). Earlier, Hui and colleagues isolated several triterpenoids from *R. tomentosa* leaves including lupeol, β-amyrin, β-amyrenonol, and botulin [7] that have potential inhibitory activity against human oxidosqualene cyclase [8]. The repetition of an investigation of the petrol extracts of *R. tomentosa* led to the isolation of the new triterpenoid, 3β-hydroxy-21α-hop-22(29)-en-30-al [9]. Likewise, various terpenoids such as taraxerol, betulin, botulin-3-acetate, 3*β*-acetoxy-11*α*-epoxyoleanan-28,13*β*-olide, 3*β*-acetoxy-12*α*-hydroxyoleanan-28,13*β*-olide, and 3*β*-acetoxy-12-oxo-oleanan-28,13*β*-olide were also identified in *R. tomentosa* stems [7,9]. Recently, numerous terpenoids were reported from *R. tomentosa* leaves, such as rhodomentones A and B [10], tomentosenol A, 4S-focifolidione, 4R-focifolidione [11], tomentodione E [12], rhodomyrtials A and B, tomentodiones A–D [13], tomentodiones E–G, and tomentodiones H-M [14], and from *R. tomentosa* root, such as tomentodiones H–M [15]. 

The phenolic compounds were also identified as the major component in the *R. tomentosa* [16,17]. According to Hiranrat and Mahabusarakam [18], the acetone extract of *R. tomentosa* leaves contains four new types of acylphlorogucinols including rhodomyrtosone A, rhodomyrtosone B, rhodomyrtosone C, and rhodomyrtosone D. Furthermore, Hiranrat et al. [19] have revealed a new flavellagic acid derivative, 3,3′,4,4′-tetra-O-methylflavellagic acid and six known compounds, including *trans*-triacontyl-4-hydroxycinnamate, 3-*O*-(*E*)-coumaroyloleanolic acid, (-)-(2 *R*,3 *R*)-1,4-*O*-diferuloylsecoisolariciresinol, arjunolic acid, 4-hydroxy-3-methoxybenzoic acid, and gallic acid, from the stems of *R. tomentosa*. Moreover, a new phloroglucinol, named rhodomyrtosone I, and six known compounds, including stigmast-4-en-3-one, rhodomyrtone, rhodomyrtosone D, oleanolic acid, methyl gallate, and 3-*O*-methylellagic acid 4-*O*-rhamnopyranoside, were also identified. In another study, Hiranrat and colleagues have isolated two phloroglucinols named tomentosones A and B from the CH_2_Cl_2_ extract of *R. tomentosa* leaves and two new phloroglucinols named rhodomyrtosones G and H from the crude hexane extract of *R. tomentosa* leaves [20,21]. Additionally, seven undescribed phloroglucinol derivatives, tomentodiones N−T from *R. tomentosa* leaves [22], and watsonianone A [23] from *R. tomentosa* fruits have been reported. On the other hand, Lowry [24] and He et al. [25] have identified different anthocyanins in the *R. tomentosa* flower such as malvidin-3-glucoside, pelargonidin-3,5-biglucoside, delphinidin-3-galactoside, and cyanidin-3-galactoside. Furthermore, various flavone glycosides such as myricetin 3-O-α-L-furanoarabinoside, myricetin 3-O-β-D-glucoside, and myricetin 3-O-α-L-rhamnoside were also found in *R. tomentosa* leaves [26]. Notably, rhodomyrtone, an antibiotic agent, and piceatannol 4′-O-β-D-glucopyranoside, a skin cosmetic agent, were determined in *R. tomentosa* leaves [27,28]. In 2004, Fahmi and colleagues have reported the purification of the flavonoid compound, combretol, from the bark and twigs of *R. tomentosa* [29]. In 2007, Phan and colleagues have studied kaempferol 3-*O-β*-sambubioside in *R. tomentosa* buds [30]. In addition, various hydrolysable tannins were also isolated from the leaves of *R. tomentosa*, such as tomentosin [31], pedunculagin, casuariin, and castalagin [32]. Some essential oils, such as α-pinene, β-pinene, and aromadendrene, were also found from *R. tomentosa* leaves [33].

## 5. Genetic Diversity

According to Hamrick and Godt [34], the genetic diversity within and among plant populations can be considerably affected by their breeding systems. Hue and colleagues have revealed that the 15 populations of *R. tomentosa* from Malaysia contain a relatively high level of genetic diversity (The total population gene diversity = 0.2510; Shannon information index = 0.3897; percentage of polymorphic bands = 95.29%) by using inter-simple sequence repeat (ISSR) markers [35]. A high level of genetic differentiation (genetic differentiation between populations = 0.6534) and a low level of gene flow (Nm = 0.2652) was also seen among the *R. tomentosa* populations. Likewise, Yao [36] has investigated the genetic diversity of *R. tomentosa* using ISSR markers. A total of 300 individuals from 10 natural populations in Hong Kong were studied with 11 ISSR primers in genetic diversity analysis. It was revealed that a high level of genetic variation was observed at the species level. The coefficient of genetic differentiation among populations was relatively high and the genetic flow was low compared to other outcrossing species. *R. tomentosa* has a wide range of distribution across the Southeast Asian region, as well as across some of the East Asian region. *R. tomentosa* was once growing profusely with somewhat contiguous large population sizes, contributing to its large gene pool with abundant genetic diversity. Recently, although its habitats have become fragmented due to anthropogenic disturbances and populations, its high variability has been and continues to be well conserved in these severely isolated populations, thus leading to the high level of genetic diversity among the populations [35].

## 6. Medicinal Uses

*R. tomentosa* has been used as traditional medicine for a long time in Asian countries such as China, Vietnam, Indonesia, Thailand, and Malaysia. The native people in Malaysia use the berries as a remedy for dysentery and diarrhea [37]. Parts of the roots and trunk are used for stomach ailments and as a traditional medicine for postpartum women. The local people of Indonesia have been using the crushed leaves of *R. tomentosa* to treat wounds. In Thailand, *R. tomentosa* is used as antipyretic, antidiarrheal, and antidysentery medicine [38]. In China, *R. tomentosa* is used for the treatment of urinary tract infections. Moreover, *R. tomentosa* is used as a traditional medicine for the treatment of pain, heartburn, and snake bites in Singapore [39]. Meanwhile, the *R. tomentosa* fruits have been used to treat diarrhea and dysentery, and to boost the immune system in Vietnam [40]. In addition to being used in folk medicine, *R. tomentosa* fruits are used to make a famous fermented drink called “Ruou Sim” at Phu Quoc Island, in the south of Vietnam. Cultivation of *R. tomentosa* to harvest fruits and to produce “Ruou sim” is done in Phu Quoc Island and extends to many provinces in the south and center of Vietnam.

## 7. Optimal Conditions for Active-Component Extraction

According to Wu and colleagues, the optimal conditions for spray drying purified flavonoid extract from *R. tomentosa* fruits were investigated by response surface methodology [41]. The optimized condition for microencapsulation was indicated with a maltodextrin to gum Arabic ratio of 1:1.3, total solid content of 27.4%, glycerol monostearate content of 0.25%, and a core to coating material ratio of 3:7, resulting in a flavonoid extract of 91.75% purity. Prepared at the optimized conditions, the flavonoid extract microcapsules were irregular spherical particles with low moisture content (3.27%), high solubility (92.35%), and high bulk density (0.346 g/cm^3^) [41]. Le et al. [42] investigated the effect of two technical parameters, namely core/wall ratio and inlet temperature of the drying agent, on the retention of antioxidants in *R. tomentosa* fruit powder during the drying process. It was observed that a decrease in the core/wall ratio from 1:4 to 1:5 reduced the antioxidant retention due to high viscosity of the feed solution. The inlet temperature of the drying agent was augmented from 150 to 180 °C leading to a decrease in moisture content and an increase in the retention of antioxidants. Meanwhile, an increase in inlet temperature from 180 to 190 °C had a detrimental effect on antioxidant retention during the spray drying of fruit juice [42]. In addition, ultrasonic treatment significantly improved both antioxidant content and activity of the extract. The optimal ultrasonic power and time were 25 W/g and 6.5 min, respectively, under which the concentration of total phenolics and ascorbic acid in the extract reached 6067 mg gallic acid equivalent/L and 516 mg/L, respectively. Furthermore, the extract obtained under the conditions of 65% ethanol, 45 °C, and 30 min exhibited high total polyphenol content (976.42 mg Gallic acid equivalent/g dry weight) and antioxidant capacity (1408.99 µM Trolox equivalents/g dry weight) [43]. 

Likewise, Le et al. [44] have optimized the extract conditions for achieving a high content of the total phenolic compound (TPC) from *R. tomentosa* fruits. The optimal conditions of extraction were suggested to be 100% methanol, 3 h of extraction time at a temperature of 40 °C, and a solvent-to-solid ratio of 2/1 (*v*/*w*). Zhao et al. [45] have estimated the effects of three thermal drying methods, namely hot air drying (HD), microwave drying (MD), and combined microwave–hot-air-drying (CD), on the phenolic profiles and antioxidant activity of *R. tomentosa* fruits. It was found that the total phenolic, flavonoid, and anthocyanin contents of CD fruits were significantly higher than those of HD and MD fruits. CD fruits had higher contents of individual phenolics and showed stronger antioxidant activity than HD and MD fruits. Thus, the CD method was suggested as a drying technique of *R. tomentosa* fruits to maintain their phenolics and antioxidant activity. Liu et al. [46] have optimized the extraction of anthocyanins from freeze-dried fruit skin of *R. tomentosa* using response surface methodology. The optimal conditions for maximum yields of anthocyanin (4.358 ± 0.045 mg/g) were 60% ethanol containing 0.1% (*v*/*v*) hydrochloric acid, 15.7:1 (*v*/*w*) liquid to solid ratio, at 64.38 °C with a 116.88 min extraction time. Furthermore, the extraction of piceatannol from the *R. tomentosa* fruits was also optimized [47]. The optimized conditions were suggested to be 78.8% ethanol, 85.3 °C, and an extraction time of 78.8 min. 

## 8. Pharmaceutical Properties

### 8.1. Anti-Inflammatory Activities

Inflammation is associated with a large range of mediator productions and releases that initiate the inflammatory response, recruit, and activate other cells to the site of inflammation [48]. Excessive or prolonged inflammation can prove harmful, contributing to the pathogenesis of a variety of diseases [49]. Herein, Jeong and colleagues have determined the anti-inflammatory activity of *R. tomentosa* in vitro for the first time [50]. It was revealed that the methanol extract from the leaves of this plant (Rt-ME) clearly inhibited the production of NO and prostaglandin E2 in lipopolysaccharide-activated RAW264.7 cells and peritoneal macrophages. The inhibitory effect of Rt-ME was due to suppressing the activation of both nuclear factor (NF)-κB and activator protein (AP)-1 pathways by directly targeting Syk/Src and IRAK1/IRAK4. Moreover, rhodomyrtone, a member of the acylphloroglucinols isolated from *R. tomentosa* leaves was determined to suppress TNF-α expression in monocytes stimulated with heat-killed methicillin-resistant *Staphylococcus aureus* (MRSA) [51]. Treatment with rhodomyrtone also significantly up-regulated the expression of the key pattern-recognition receptors, TLR2 and CD14, in THP-1 monocytes, contributing to the elimination of MRSA from the monocytes. Notably, the 80% ethanol extract and piceatannol from *R. tomentosa* fruits reduced UVB-induced cytotoxicity and inflammatory mediator production of prostaglandin E2 in normal human epidermal keratinocytes [52]. These results indicated that *R. tomentosa* fruit extract and its key constituent, piceatannol, are potential candidates for the treatment of UV-induced skin inflammation.

Recently, the acylphloroglucinol rhodomyrtone from *R. tomentosa* leaves was evidenced as a potential inhibitor of inflammation. The co-exposure of rhodomyrtone with LPS resulted in a prominent down-regulation in the expression of inflammatory-process-related genes including *IL-1β, IL-8, TNF-α, iNOS, SAA*, and *Hepcidin* and reduction in cellular reactive oxygen species (ROS) levels by head kidney macrophages [53]. Likewise, Zhang and colleagues have further determined that phloroglucinol derivatives from *R. tomentosa* leaves possessed the anti-inflammatory activity via decreasing the NO production from LPS-induced RAW 264.7 cells with the half maximal inhibitory concentration (IC_50_) values of 3.8–74.3 μM [24]. Especially, rhodomyrtone from *R. tomentosa* leaves was capable of inhibiting the transcription and expression of a number of inflammatory mediators (*DEFB4, IL1B, IL17C, IL36G, LCN2, PI3, S100A7,* and *S100A8* transcripts) from TNF-α- and IL-17A-stimulated skin organ cultures, via suppression of NF-κB, ERK, JNK, and p38 signaling pathways. Moreover, it attenuated imiquimod-induced skin inflammation in mice. The data supported the efficacy of rhodomyrtone for treating psoriasis through the inhibition of keratinocyte hyperproliferation [54]. These results indicate that *R. tomentosa* and its components exert anti-inflammatory effects that open up the possibility of using these natural products for further development of health beneficial products regarding prevention and/or treatment of inflammation. 

### 8.2. Antioxidant Activity

Oxidative stress causes more than a hundred types of human diseases due to peroxidation of membrane lipids, protein modification, depletion of nicotinamide nucleotides, cytoskeletal disruption, and DNA damage [55]. The high-antioxidant agents from natural products can play an important role in the prevention and treatment of free-radical-caused diseases [56]. Among such natural products, *R. tomentosa* has been determined as an effective antioxidant agent (Table 2). According to Lavanya et al. [57], *R. tomentosa* leaves extract significantly inhibited the generation of lipid peroxides. The lipid peroxidation inhibition capacity of the extract was equal to 0.93 ± 0.07 mM gallic acid at 100 μg/mL. The extract showed a rapid and increased tendency to reduce Fe^3+^ to Fe^2+^, equivalent to 10.8 ± 1.12 mM gallic acid and 30.5 ± 5.22 mM ellagic acid, respectively, at 1 mg/mL. Moreover, *R. tomentosa* extract exhibited protective effects against CCl_4_-induced decrease in SOD, CAT, and GPx enzyme activities in blood, liver, and kidneys. At the dose of 0.8 g/kg body weight, the recovery of enzyme activities were significant and similar to the effect of α-tocopherol (0.1 g/kg body weight). On the other hand, the fruit extract exhibited 62.13% DPPH scavenging activity at a concentration of 200 µg/mL with 36% metal chelating ability at a concentration of 100 µg/mL [58]. 

The antioxidant activity of *R. tomentosa* fruit extract was suggested to be due to phenolic compounds. Indeed, it was determined that the purified anthocyanin extract from the fruits of *R. tomentosa* possessed strong antioxidant activities, including DPPH radical-scavenging capacity (IC_50_, 6.27 ± 0.25 µg/mL), ABTS radical-scavenging capacity (IC_50_, 90.3 ± 1.52 µg/mL), and oxygen radical-absorbance capacity (IC_50_, 9.29 ± 0.08 µmol TE/mg) [59]. Moreover, piceatannol is also known as a strong antioxidant agent due to hydroxyl groups in its stilbene rings [60]. Notably, piceatannol was the major phenolic compound in *R. tomentosa* fruits with a concentration of 2.3 mg/g dry weight at the full maturity stage. Therefore, it may contribute to the antioxidant ability of *R. tomentosa* fruits. Likewise, the in vitro and in vivo antioxidant activities of the flavonoid-rich extract from *R. tomentosa* fruits were confirmed via their reducing power (EC_50_, 28.67 ± 1.37 µg/mL), scavenging superoxide radicals (EC_50_, 214.83 ± 6.54 µg/mL), hydroxyl radicals (EC_50_, 217.73 ± 3.46 µg/mL), and DPPH radicals (EC_50_, 10.97 ± 0.18 µg/mL), as well as by inhibiting lipid peroxidation effectively. The flavonoid-rich extract significantly enhanced the activities of antioxidant enzymes such as SOD, GSH-Px, and CAT in serums of mice after they were administered the extract [61]. 

### 8.3. Antimicrobial Activity

Bacteria are becoming resistant to clinically used drugs and the discovery of new antibiotics to fight against resistant bacterial species is always necessary. *R. tomentosa* have a strong antimicrobial activity and valuable medical potential to be developed into an effective drug (Table 3). The fruit and leaf extract of *R. tomentosa* exhibited such activities against *Bacillus cereus* and *Candida albicans* with AI (AI, activity index, is the zone of inhibition of extract/the zone of inhibition of chloramphenicol) of 0.42 and 0.35, respectively. Leaves, stem, twig, and fruit of the plant showed activity against *Salmonella typhi* and *Propionibacterium acnes* with an AI of 0.19–0.50 in comparison to that of the reference compound, chloramphenicol [62]. The ethanol extract of *R. tomentosa* leaves had profound antibacterial activity against all staphylococcal bacteria isolated from milk with the minimum inhibitory concentration (MIC) and minimum bactericidal concentration (MBC) values ranged from 16 to 64 μg/mL and from 64 to128 μg/mL, respectively [63]. It also exhibited antibacterial activity against *S. aureus* ATCC 25923, *Streptococcus mutans*, and *C. albicans* ATCC 90028 with MIC values of 31.25, 15.62, and 1000 µg/mL, respectively [64]. Moreover, this extract effectively inhibited *Streptococcus agalactiae* and *Streptococcus iniae* isolated from infected tilapia with MIC values ranging from 7.8 to 62.5 µg/mL. The pretreated cells caused a significant reduction in the mortality of *S. agalactiae*-infected Nile tilapia [65]. Furthermore, the clinical isolates of *Streptococcus pyogenes* were also inhibited by this extract with an MIC value range of 3.91–62.5 μg/mL [66]. Notably, the surviving cells were not detected after 16 h of treatment with 8 × MIC of the extract. It was determined that the antibacterial activity was not due to the lysis and cytoplasmic leakage of the bacterial membrane. 

Likewise, Rosli and colleagues have shown that the methanol extract of *R. tomentosa* possessed strong inhibition properties against *Escherichia coli* and *S. aureus* with an inhibition zone of 10 mm each for leaves, 16 and 12 mm for fruits, and 10 and 13 mm for stems, respectively [67]. In addition, *R. tomentosa* ethanolic leaf extract has been evidenced as a biocontrol agent against *Listeria monocytogenes* [68] and *E. coli* O157:H7 [69], an important foodborne pathogen implicated in many outbreaks of listeriosis. The MIC and MBC values ranged from 16 to 32 µg/mL and from 128 to 512 µg/mL, respectively [68]. As a result, *R. tomentosa* leaf extract has potential for further development into a biocontrol agent in food to prevent the incidence of contamination. 

The compounds from *R. tomentosa* have also been isolated and evaluated for antibacterial activities. Rhodomyrtone has been evidenced as a new candidate as a natural antibacterial drug from *R. tomentosa*. Rhodomyrtone displayed significant antibacterial activities against Gram-positive bacteria including *B. cereus, Bacillus subtilis, Enterococcus faecalis, S. aureus,* methicillin-resistant *S. aureus* (MRSA), *Staphylococcus epidermidis, Streptococcus gordonii, S. mutans, Streptococcus pneumoniae, S. pyogenes, Streptococcus salivarius*, *Clostridium difficile* [70,71,72,73], and antibiotic-resistant pathogens including epidemic methicillin-resistant *S. aureus*, vancomycin-intermediate *S. aureus*, and vancomycin-resistant enterococcal strains [74]. The minimum bactericidal concentration (MBC) of rhodomyrtone ranged from 0.39 to 0.78 µg/mL [70]. Moreover, rhodomyrtone effectively inhibited *P. acnes* with an MIC_90_ value of 0.5 µg/mL. The numbers of the bacterial cells were reduced by at least 99% after treatment with rhodomyrtone within 24 h [75]. Especially, rhodomyrtone was observed to be able to prevent biofilm formation and to kill mature biofilms of *S. mutans* [76], *S. aureus*, *S. epidermidis* [77], and *P. acnes* [78]. 

Up to now, numerous studies regarding the mechanism of action of rhodomyrtone as a natural antibacterial agent have been reported. According to Leejae et al. [79], rhodomyrtone inhibited staphyloxanthin biosynthesis in bacteria, and thus increased the susceptibility of the pathogen to H_2_O_2_ and singlet oxygen killing. According to Bach et al. [76], rhodomyrtone suppressed acid production from bacteria by inhibiting enzyme activities responsible for acid production and tolerance, including membrane-bound enzymes F-ATPase and phosphotransferase system, as well as glycolysis enzymes glyceraldehyphosphate dehydrogenase and pyruvate kinase in cytoplasm. Limsuwan and colleagues have found that the antibacterial activity of rhodomyrtone was due to interference in metabolic pathways such as glycolysis, gluconeogenesis, and amino acid metabolism, and inhibiting the expression of streptococcal toxins such as the CAMP factor and streptococcal pyrogenic exotoxin C [72]. 

Visutthi and colleagues have suggested that the antibacterial activity of rhodomyrtone was due to the suppression of staphylococcal antigenic proteins, immunodominant antigen A, and staphylococcal secretory antigen involved in cell wall hydrolysis, and disturbing the bacterial cell wall biosynthesis [80] and cell division [81]. It caused prominent changes including alterations in cell wall, abnormal septum formation, cellular disintegration, and cell lysis [81]. Moreover, Mitsuwan and colleagues have revealed that rhodomyrtone altered enzymes and metabolites involved in several metabolic pathways including amino acid biosynthesis, nucleic acid biosynthesis, and glucid and lipid metabolism. The levels of two enzymes (glycosyltransferase and UTP-glucose-1-phosphate uridylyltransferase) and three metabolites (UDP-glucose, UDP-glucuronic acid, and UDP-N-acetyl-D-galactosamine) participating in the synthesis of the pneumococcal capsule clearly diminished in the bacterial cells exposed to rhodomyrtone [82]. Additionally, Sianglum et al. [83] have provided relevant data to clarify that rhodomyrtone is a bacterial cell membrane-damaging agent. Notably, Saeloh et al. [84] have demonstrated that rhodomyrtone caused large membrane invaginations with a dramatic increase in fluidity, which attracted a broad range of membrane proteins and trap proteins. Furthermore, molecular dynamics simulations showed that rhodomyrtone transiently binds to phospholipid head groups and causes distortion of lipid packing, providing explanations for membrane fluidization and induction of membrane curvature. Both its transient binding mode and its ability to form protein-trapping membrane vesicles are unique, making it an attractive new antibiotic candidate with a novel mechanism of action [84].

### 8.4. Anticancer Activity

Cancer can be defined as a disease in which a group of abnormal cells grow uncontrollably by disregarding the normal rules of cell division. Cancer continues to be one of the major causes of death worldwide and mortality levels have increased every year [85]. Typical antitumoral therapies such as surgery, chemotherapy, and radiotherapy have been subject to some improvements. However, the use of these therapies does not show satisfying results, and even causes side effects [86]. A promising approach is associated with natural products that are available as chemoprotective agents against commonly occurring cancers worldwide [87,88,89]. Among them, *R. tomentosa* has been reported as a promising natural anticancer agent (Table 4). The ethyl acetate extract of *R. tomentosa* roots showed significant anti-proliferative activity on HepG2 (IC_50_ = 11.47 ± 0.280 µg/mL), MCF-7 (IC_50_ = 2.68 ± 0.529 µg/mL), and HT29 (IC_50_ = 16.18 ± 0.538 µg/mL) after 72 h of treatment [90]. Moreover, rhodomyrtone from *R. tomentosa* leaves was able to suppress, 13.62–61.61%, 50.59–80.16%, and 61.82–85.34%, HaCaT cell proliferation at concentrations of 2–32 µg/mL after 24, 48, and 72 h treatments, respectively. HaCaT keratinocytes treated with rhodomyrtone showed chromatin condensation, fragmentation of nuclei, and induction of apoptosis. Flow cytometric analysis demonstrated an increase in the percentage of apoptosis (1.2–10%, 8.2–35.4%, and 21.0–77.8%) of keratinocytes after 24, 48, and 72 h treatments of rhodomyrtone (2–32 µg/mL), respectively [91] Moreover, rhodomyrtone inhibited the proliferation of human epidermoid carcinoma A431 cells with an IC_50_ value of 8.04 ± 0.11 µg/mL. It increased chromatin condensation, nuclear fragmentation, and apoptotic bodies in the treated cells, induced cell apoptosis through the activation of caspase-7 and poly (ADP-Ribose) polymerase cleavage, and caused cell cycle arrest at the G1 phase. Notably, the nontoxic concentration of rhodomyrtone markedly inhibited A431 cell migration in a dose- and time-dependent manner [92]. 

Likewise, Tayeh and colleagues have also reported that rhodomyrtone (0.5 and 1.5 µg/mL) exhibited pronounced inhibition on A431 cancer cell metastasis by reducing cell migration, cell adhesive ability, and cell invasion. Herein, the inhibitory activity of rhodomyrtone on A431 cell metastasis was identified via suppressing ERK1/2, p38, NF-κB, and FAK/Akt signaling pathways, and thus reducing matrix metallopeptidase (MMP)-2/9 activities and expression [93]. On the other hand, several active phloroglucinol derivatives from *R. tomentosa* leaves including rhodomyrtosone I and rhodomyrtosone B exhibited obvious inhibitory activities on HeLa and Vero cells with IC_50_ values < 10 μM [19]. Piceatannol has been reported to induce apoptosis and cell cycle arrest in human melanoma cells [94] and hepatoma cells [95]. Especially, piceatannol was revealed as the major phenolic compound in *R. tomentosa* fruits that is 1000–2000 times higher than that of red grapes [28]. Therefore, piceatannol is considered to be an important component that significantly contributes to the anticancer activity of *R. tomentosa*. Recently, Zhou and colleagues have found that tomentodione M, a novel meroterpenoid isolated from *R. tomentosa* leaves, increased the cytotoxicity of chemotherapeutic drugs such as docetaxel and doxorubicin in human breast cancer cells/reversed multidrug resistance (MCF-7/MDR cells) and human immortalized myelogenous leukemia cells/reversed multidrug resistance (K562/MDR cells). Additionally, the anticancer activity of tomentodione M was observed due to reducing colony formation, enhancing apoptosis in docetaxel-treated MCF-7/MDR and K562/MDR cells, increasing intracellular accumulation of doxorubicin and rhodamine 123 in MDR cancer cells, and down-regulating P-gp mRNA and protein expression [96]. Thus, tomentodione M may be a useful anticancer natural product. 

### 8.5. Other Biological Activities

Chai et al. [97] evaluated the antidepressant effects of rhodomyrtone from *R. tomentosa* leaves in mice with chronic unpredictable mild stress-induced depression. Rhodomyrtone possessed a protective effect against depression-like behaviors via preventing source consumption decrease and decreased social behaviors. Rhodomyrtone prevented the impairment of spatial memory, reversed dendritic spine density defects, inhibited the increase of glycogen synthase kinase-3β activity, and reversed the decrease of brain-derived neurotrophic factor and postsynaptic density protein 95 in chronic unpredictable mild stress mice. Moreover, the elevated expression of apoptosis-associated protein Bax and cleaved-caspase 3 was also reversed by rhodomyrtone treatment. 

Maskam et al. [58] determined the preventive effect of *R. tomentosa* fruit extracts against the formation of atherosclerosis in New Zealand white rabbits. It was observed that total cholesterol, low-density lipoprotein, and lipid peroxidation were significantly reduced and high-density lipoprotein and triacylglycerides were markedly increased in rabbits fed with a cholesterol 1% diet and fruit extract 50 mg/kg as compared with the group on cholesterol 1% diet alone. 

The anti-diabetic activity of *R. tomentosa* aqueous leaf extract was also reported by Hasibuan and colleagues [98]. The administration of aqueous extract resulted in the lowering of blood sugar levels in alloxan-induced diabetic mice at the dose of 100 mg/kg.

The role of anthracene glycosides from *R. tomentosa* in bone formation was observed via stimulating the process of osteoblastic differentiation [99]. The osteoblast differentiation was assessed by measuring the alkaline phosphatase activity. The treatment of compound 4, 8, 9, 10-tetrahydroxy-2, 3, 7-trimethoxyanthracene-6-O-β-D-glucopyranoside and 2, 4, 7, 8, 9, 10-hexahydroxy-3-methoxyanthracene-6-O-α-L-rhamnopyranoside significantly increased the alkaline phosphatase activity, collagen synthesis, and mineralization of the nodules of MC3T3-E1 osteoblastic cells. Their therapeutic potentials as a new agent for osteoporosis should be explored by further studies.

According to Geetha et al. [100], the anti-ulcerogenic activity of an aqueous alcoholic (70%) extract of *R. tomentosa* was investigated using acetic-acid-induced chronic ulcer model in rats. The antiulcer activity was indicated by the reduction in ulcer index, the increase in the levels of superoxide dismutase and catalase, and the decrease in lipid peroxidation. It was suggested that the presence of triterpenoids, flavonoids, and phenolic compounds is probably related to the potent anti-ulcerogenic activity. 

## 9. Conclusions

Accordingly, *R. tomentosa* has different nutritional compositions such as proteins, amino acids, carbohydrates, lipids, fatty acids, minerals, and vitamins. Moreover, *R. tomentosa* is a promising source of biologically active metabolites including phenolic and terpenoid compounds. Notably, various health beneficial effects of *R. tomentosa* including antioxidant, antibacterial, anti-inflammatory, and anticancer activities have been revealed by in vitro and in vivo experimental models. Thus, it is believed that *R. tomentosa* can be applied as a functional food for prevention and/or treatment of chronic diseases. However, further studies regarding the discovery of novel compounds and biological activities of *R. tomentosa* and the development of new health benefit products are necessary in the future. 

## Figures and Tables

**Table 1:** Phytochemical composition of *Rhodomyrtus tomentosa.*

No.	Compounds	Classification	Source	Ref.
1	Lupeol, β-amyrin, β-amyrenonol, and botulin	Terpenoid	Leaves	[7]
2	3β-hydroxy-21α-hop-22(29)-en-30-al	Terpenoid	Leaves	[9]
3	Rhodomentones A and B	Terpenoid	Leaves	[10]
4	Tomentosenol A, 4S-focifolidione, and 4R-focifolidione	Terpenoid	Leaves	[11]
5	Tomentodione E	Terpenoid	Leaves	[12]
6	Rhodomyrtials A and B, tomentodiones A–D	Terpenoid	Leaves	[13]
7	Tomentodiones E–G and tomentodiones H–M	Terpenoid	Leaves	[14]
8	Tomentodiones H–M	Terpenoid	Roots	[15]
9	Rhodomyrtosone A, rhodomyrtosone B, rhodomyrtosone C, and rhodomyrtosone D	Phenolics	Leaves	[18]
10	3,3′,4,4′-tetra-O-methylflavellagic acid, rhodomyrtosone I, stigmast-4-en-3-one, rhodomyrtone, rhodomyrtosone D, oleanolic acid, methyl gallate, and 3-*O*-methylellagic acid 4-*O*-rhamnopyranoside	Phenolics	Stems	[19]
11	Tomentosones A and B, rhodomyrtosones G and H	Phenolics	Leaves	[20,21]
12	Tomentodiones N−T	Phenolics	Leaves	[22]
13	Watsonianone A	Phenolics	Fruits	[23]
14	Malvidin-3-glucoside, pelargonidin-3,5-biglucoside, delphinidin-3-galactoside, and cyanidin-3-galactoside	Phenolics	Flowers	[24,25]
15	Myricetin 3-O-α-L-furanoarabinoside, myricetin 3-O-β-D-glucoside, and myricetin 3-O-α-L-rhamnoside	Phenolics	Leaves	[26]
16	Rhodomyrtone and piceatannol 4′-O-β-D-glucopyranoside	Phenolics	Leaves	[27,28]
17	Combretol	Phenolics	Bark and twigs	[29]
18	Kaempferol 3-*O*-*β*-sambubioside	Phenolics	Buds	[30]
19	Tomentosin, pedunculagin, casuariin, and castalagin	Phenolics	Leaves	[31,32]
20	α-pinene, β-pinene, and aromadendrene	Lipids	Leaves	[33]

**Table 2:** The antioxidant effect of *R. tomentosa.*

No.	Bioactive Agents	Biological Activity	Ref.
1	Acetone leaves extract	Inhibiting lipid peroxidation (equal to 0.93 ± 0.07 mM gallic acid at 100 μg/mL). Reducing Fe^3+^ to Fe^2+^ (equal to 10.8 ± 1.12 mM gallic acid at 1 mg/mL) Increasing SOD, CAT, and GPx enzyme activities in blood, liver, and kidneys (0.8 g/kg body weight)	[57]
2	Methanol fruits extract	Scavenging 62.13% DPPH (200 µg/mL) and chelating 36% metal (100 µg/mL)	[58]
3	Anthocyanin extract from fruits	Scavenging DPPH (IC_50_, 6.27 ± 0.25 µg/mL) and ABTS (IC_50_, 90.3 ± 1.52 µg/mL) radicals	[59]
4	Flavonoid-rich extract from fruits	Reducing power (EC_50_, 28.67 ± 1.37 µg/mL), scavenging superoxide radicals (EC_50_, 214.83 ± 6.54 µg/mL), hydroxyl radicals (EC_50_, 217.73 ± 3.46 µg/mL), and DPPH radicals (EC_50_, 10.97 ± 0.18 µg/mL), and inhibiting lipid peroxidation Enhancing SOD, GSH-Px, and CAT in serums of mice	[61]

**Table 3:** The antimicrobial activities of *R. tomentosa.*

No.	Bioactive Agents	Biological Activity	Ref.
1	Leaves, stem, twig and fruit extracts	Inhibiting *Bacillus cereus, Candida albicans, Salmonella typhi*, and *Propionibacterium acnes*	[62]
2	Ethanol leaves extract	Inhibiting staphylococcal bacteria from milk, MIC = 16–64 μg/mL and MBC = 64–128 μg/mL	[63]
3	Ethanol leaves extract	Inhibiting *Staphylococcus aureus* ATCC 25923, *Streptococcus mutans*, and *C. albicans* ATCC 90028, MIC = 31.25, 15.62, and 1000 µg/mL, respectively.	[64]
4	Ethanol leaves extract	Inhibiting *Streptococcus agalactiae* and *Streptococcus iniae*, MIC = 7.8–62.5 µg/mL	[65]
5	Ethanol leaves extract	Inhibiting *Streptococcus pyogenes*, MIC = 3.91–62.5 μg/mL	[66]
6	Methanol extracts of leaves, fruits, and stems	Inhibiting *Escherichia coli* and *S. aureus*	[67]
7	Ethanol leaves extract	Inhibiting *Listeria monocytogenes*, MIC = 16–32 µg/mL and MBC = 128–512 µg/mL	[68]
8	Rhodomyrtone	Inhibiting *B. cereus, Bacillus subtilis, Enterococcus faecalis, S. aureus,* methicillin-resistant *S. aureus* (MRSA), *Staphylococcus epidermidis, Streptococcus gordonii, S. mutans, Streptococcus pneumoniae, S. pyogenes, Streptococcus salivarius*, *Clostridium difficile*, epidemic methicillin-resistant *S. aureus*, vancomycin-intermediate *S. aureus*, and vancomycin-resistant enterococcal strains, MBC = 0.39–0.78 µg/mL	[70,71,72,73,74]
9	Rhodomyrtone	Inhibiting staphyloxanthin biosynthesis in bacteria	[79]
10	Rhodomyrtone	Suppressing acid production and tolerance via inhibiting membrane-bound enzymes F-ATPase and phosphotransferase system, glyceraldehyphosphate dehydrogenase, and pyruvate kinase	[76]
11	Rhodomyrtone	Interfering in metabolic pathways such as glycolysis, gluconeogenesis, and amino acid metabolism and inhibiting the expression of streptococcal toxins such as the CAMP factor and streptococcal pyrogenic exotoxin C	[72]
12	Rhodomyrtone	Suppressing cell wall hydrolysis, disturbing the bacterial cell wall biosynthesis and cell division	[80,81]
13	Rhodomyrtone	Inhibiting amino acid biosynthesis, nucleic acid biosynthesis, and glucid and lipid metabolism	[82]
14	Rhodomyrtone	Causing bacterial cell membrane damage and membrane invaginations	[83,84]

MBC: Minimum bactericidal concentration.

**Table 4:** The anticancer activity of *R. tomentosa.*

No.	Bioactive Agents	Biological Activity	Ref.
1	Ethyl acetate extract of roots	Anti-proliferative activity on HepG2 (IC_50_ = 11.47 ± 0.280 µg/mL), MCF-7 (IC_50_ = 2.68 ± 0.529 µg/mL), and HT29 (IC_50_ = 16.18 ± 0.538 µg/mL) after 72 h.	[90]
2	Rhodomyrtone	Suppressing 61.82–85.34% HaCaT cell proliferation after 72 h treatment. Inducting 21.0–77.8% apoptosis of keratinocytes after 72 h treatment.	[91]
3	Rhodomyrtone	Inhibiting proliferation of human epidermoid carcinoma A431 cells (IC_50_ = 8.04 ± 0.11 µg/mL). Inducing cell apoptosis through the activation of caspase-7 and poly (ADP-Ribose) polymerase cleavage, and causing cell cycle arrest at the G1 phase.	[92]
4	Rhodomyrtone	Inhibiting A431 cancer cell metastasis by reducing cell migration, cell adhesive ability, and cell invasion.	[93]
5	Rhodomyrtosone I and B	Inhibiting HeLa and Vero cells (IC_50_ < 10 μM)	[19]
6	Piceatannol	Inducing apoptosis and cell cycle arrest in human melanoma cells and hepatoma cells.	[94,95]
7	Tomentodione M	Increasing the cytotoxicity of chemotherapeutic drugs in human breast cancer cell/reversed multidrug resistance and human immortalized myelogenous leukemia cells/reversed multidrug resistance. Enhancing cell apoptosis.	[96]
